# Targeting of the MAPK and AKT pathways in conjunctival melanoma shows potential synergy

**DOI:** 10.18632/oncotarget.10770

**Published:** 2016-07-22

**Authors:** Jinfeng Cao, Renier C. Heijkants, Aart G. Jochemsen, Mehmet Dogrusöz, Mark J. de Lange, Pieter A. van der Velden, Sjoerd H. van der Burg, Martine J. Jager, Robert M. Verdijk

**Affiliations:** ^1^ Department of Ophthalmology, Leiden University Medical Center, Leiden, The Netherlands; ^2^ Department of Ophthalmology, The Second Hospital of Jilin University, Changchun, China; ^3^ Department of Molecular Cell Biology, Leiden University Medical Center, Leiden, The Netherlands; ^4^ Department of Pathology, Section Ophthalmic Pathology, Erasmus MC University Medical Center, Rotterdam, The Netherlands; ^5^ Department of Clinical Oncology, LUMC, Leiden, The Netherlands

**Keywords:** conjunctival melanoma, MAPK, AKT, nevus, kinase inhibitor

## Abstract

**Purpose:**

Conjunctival melanoma (CM) is a rare but lethal form of cancer. Similar to cutaneous melanoma, CM frequently carries activating mutations in *BRAF* and *NRAS*. We studied whether CM as well as conjunctival benign and premalignant melanocytic lesions express targets in the mitogen-activated protein kinase (MAPK) and AKT pathways, and whether specific inhibitors can suppress CM growth *in vitro*.

**Methods:**

131 conjunctival lesions obtained from 129 patients were collected. The presence of *BRAF* V600E mutation and expression of phosphorylated (p)-ERK and p-AKT were assessed by immunohistochemistry. We studied cell proliferation, phosphorylation, cell cycling and apoptosis in three CM cell lines using two BRAF inhibitors (Vemurafenib and Dabrafenib), a MEK inhibitor (MEK162) and an AKT inhibitor (MK2206).

**Results:**

The *BRAF* V600E mutation was present in 19% of nevi and 26% of melanomas, but not in primary acquired melanosis (PAM). Nuclear and cytoplasmic p-ERK and p-AKT were expressed in all conjunctival lesions. Both BRAF inhibitors suppressed growth of both *BRAF* mutant CM cell lines, but only one induced cell death. MEK162 and MK2206 inhibited proliferation of CM cells in a dose-dependent manner, and the combination of these two drugs led to synergistic growth inhibition and cell death in all CM cell lines.

**Conclusion:**

ERK and AKT are constitutively activated in conjunctival nevi, PAM and melanoma. While BRAF inhibitors prohibited cell growth, they were not always cytotoxic. Combining MEK and AKT inhibitors led to more growth inhibition and cell death in CM cells. The combination may benefit patients suffering from metastatic conjunctival melanoma.

## INTRODUCTION

Conjunctival melanoma (CM) is a rare malignant ocular surface tumor, arising from melanocytes in the conjunctiva. Although CM accounts for 5% of all ocular melanoma, over the past decades, an increase in occurrence has been reported from Finland [1], Sweden [2] and the United States [3]. CM has a high local recurrence rate after treatment [4, 5]. Surgical excision combined with adjuvant cryotherapy, brachytherapy, and/or topical chemotherapy helps to prevent local recurrences of primary CM [5, 6]. A melanoma-related death rate of up to 29% at 10 years has been reported [5, 7]. However, the current therapeutic strategies are limited with regard to metastases.

Most CM originate in primary acquired melanosis (PAM) with atypia, while a small proportion arises from a preexisting nevus or de novo [8, 9]. The term “PAM”, created by Zimmerman [10], is used to describe the appearance of conjunctival pigmentation at some time after birth. PAM occurs with or without atypia: PAM without atypia consists of an increased amount of melanin pigment in the basal layer of the epithelium and/or melanocytic hyperplasia [11], and generally does not develop into melanoma. In contrast, PAM with atypia contains atypical melanocytic hyperplasia that can extend into the more superficial non-basal portion of the epithelium and/or contains epithelioid melanocytes [12]. Up to 71% of CM has been reported to arise from PAM with atypia, while only up to 17% of CM is associated with conjunctival nevi [5, 13].

CM shares more similarities with cutaneous melanoma than with intraocular uveal melanoma. Like cutaneous melanoma, CM frequently harbors a *BRAF* mutation [14–16], as opposed to *GNAQ/GNA11* mutations which are found in most cases of uveal melanoma. In a recent study, a *BRAF* V600E mutation was found in 29% of CM, and an *NRAS* mutation in 18% [17]. C*-KIT* mutations are seldom found in CM [18–20]. Mutant *BRAF* and *NRAS* are both known to activate the downstream kinases MEK1/2 and ERK1/2, thereby promoting tumor proliferation [21]. BRAF inhibitors (BRAFi), including Vemurafenib and Dabrafenib, can prolong survival of metastatic cutaneous melanoma patients [22, 23]. In CM, Vemurafenib has been used to target metastases and a primary melanoma with opposite outcomes: the metastatic tumor progressed after 2 months of treatment while the primary tumor was controlled for 16 months [24, 25]. MEK inhibitor (MEKi) treatment is being tested in phase II and III clinical trials of metastatic cutaneous melanoma. Recently, it was found that the PI3K/AKT signaling pathway plays a major role in the initiation, progression, invasion, and drug resistance of cutaneous melanoma [26, 27]. Overactivity of PI3K/AKT pathway can be induced by loss of activity of PTEN or by activating mutations in oncogene *NRAS*. A number of clinical trials using PI3K or AKT inhibitors (AKTi) are ongoing in patients with BRAF wild type (WT), BRAFi-resistant and NRAS-mutant cutaneous melanoma, colon cancer and ovarian carcinoma [28].

To determine whether the above-mentioned inhibitors are of use in CM, we tested the effect of several potentially useful drugs on three CM cell lines, each of which has either a *BRAF* or *NRAS* mutation. We furthermore evaluated the phosphorylation of ERK and AKT in a substantial series of conjunctival nevi, PAM without atypia, PAM with atypia and primary CM tissues.

## RESULTS

### Phosphorylation of ERK and AKT in conjunctival melanoma

**Figure 1:**
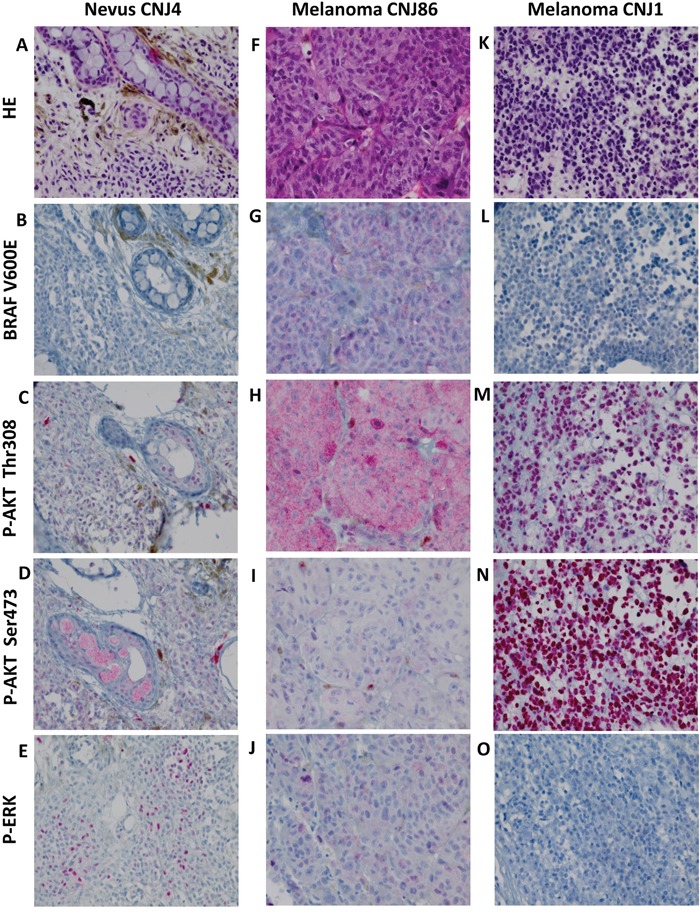
HE staining, BRAF V600E expression and phosphorylation of ERK and AKT in CM **A-E**. nevus, **F**-**J**. BRAF V600E mutated melanoma, **K**-**O**. BRAF V600E negative melanoma. HE staining (A, F, K). Positive staining for p-AKT Thr308, p-AKT Ser473 and p-ERK occurred in a cytoplasmic (H, I) and nuclear (C, M, N) fashion and in combination (D, E, J). Negative staining (B, L, O). Staining patterns for these proteins did not correlate with malignant progression. All images were taken at the magnification of ×400.

We determined the presence of *BRAF* V600E mutation in 131 pigmented conjunctival lesions from 129 patients and analyzed the expression of phosphorylated (p)-ERK and p-AKT by immunohistochemistry (Table 1). We observed *BRAF* V600E mutation in 19% of nevi (n=51) and 26% of melanoma (n=42) (Figure 1G). No *BRAF* V600E mutation was seen in any case of PAM without atypia (n=20) or PAM with atypia (n=18). One of the *BRAF* mutated melanomas evolved from a background of PAM.

**Table 1:** Frequency of positively-staining cells in an immunohistological analysis of *BRAF* V600E mutation, p-ERK and p-AKT, in conjunctival lesions

	Nevi *n*=51 (%)	PAM without atypia *n*=20 (%)	PAM with atypia *n*=18 (%)	CM *n*=42 (%)	*P* value^c^ (Nevi vs. CM)
*BRAF* V600E mutation^a^					
Negative	30 (81)	17 (100)	13 (100)	29 (74)	0.48
Positive	7 (19)	0 (0)	0 (0)	10 (26)	
P-ERK cytoplasmic expression^b^					
Absent	27 (75)	12 (75)	8 (44)	26 (72)	0.79
Present	9 (25)	4 (25)	10 (56)	10 (28)	
P-ERK nuclear expression^b^					
Absent	29 (83)	13 (81)	8 (44)	28 (78)	0.59
Present	6 (17)	3 (19)	10 (56)	8 (22)	
P-AKT Ser473 cytoplasmic expression^b^					
Absent	31 (82)	10 (83)	7 (78)	29 (74)	0.41
Present	7 (18)	2 (17)	2 (22)	10 (26)	
P-AKT Ser473 nuclear expression^b^					
Absent	9 (24)	2 (17)	3 (33)	5 (13)	0.24
Present	29 (76)	10 (83)	6 (67)	33 (87)	
P-AKT Thr308 cytoplasmic expression^b^					
Absent	12 (33)	10 (91)	8 (80)	14 (37)	0.75
Present	24 (67)	1 (9)	2 (20)	24 (63)	
P-AKT Thr308 nuclear expression^b^					
Absent	22 (61)	5 (45)	7 (70)	26 (68)	0.51
Present	14 (39)	6 (55)	3 (30)	12 (32)	

^a^ *BRAF* V600E mutation was scored as positive or negative.

^b^ The phosphorylated protein expression was evaluated according to IRS scoring system, 0-1 was regarded as absent, and 2-12 was regarded as present.

^c^ Pearson chi-square test was applied to compare nevi group versus CM group for indicated protein expression. All tests were two-sided, and *P* values of ≤0.05 were considered significant.

The presence of p-ERK and p-AKT was studied by immunohistochemistry. AKT activation is associated with phosphorylation of two residues: serine 473 (Ser473) and threonine 308 (Thr308) [29]. Using the antibody p-AKT Ser473, most p-AKT staining was observed in nuclei (Figure 1N), while the p-AKT Thr308 antibody gave both nuclear and cytoplasmic staining (Figure 1M). In all 4 groups (nevi, PAM with and without atypia, and melanoma), expression of nuclear and cytoplasmic p-ERK and p-AKT was observed. P-ERK cytoplasmic staining was seen more frequently in PAM with atypia than in nevi (*P* = 0.027) or CM (*P* = 0.046). Similarly, p-ERK nuclear expression was seen more often in PAM with atypia than in nevi (*P* = 0.004), PAM without atypia (*P* = 0.028) or CM (*P* = 0.014). In groups of nevi and CM, neither cytoplasmic nor nuclear expression was associated with the presence of a *BRAF* mutation.

### Molecular effects of Vemurafenib, Dabrafenib, MEK162 and MK2206

**Figure 2:**
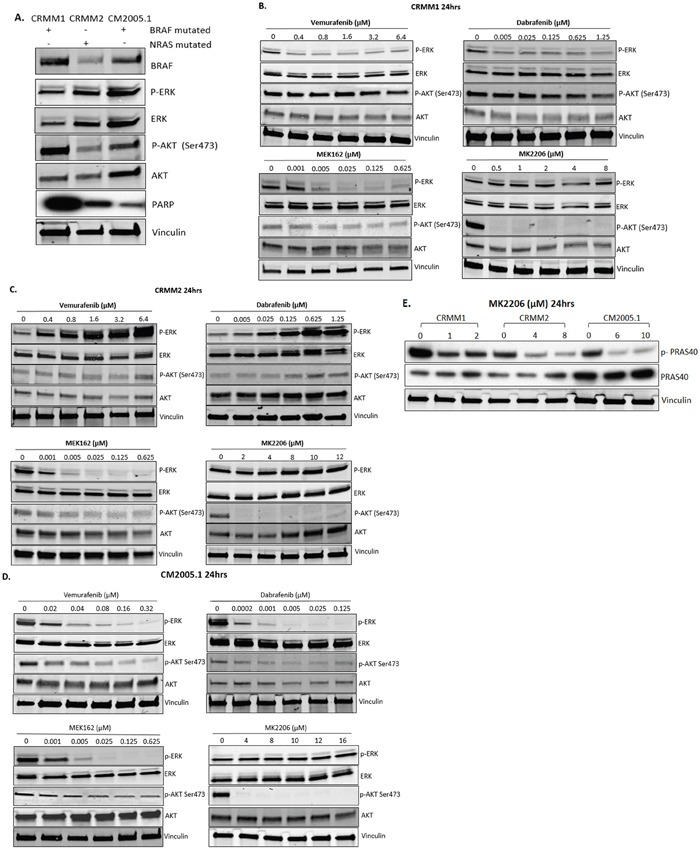
Effect of BRAFi, MEKi and AKTi treatment on ERK and AKT phosphorylation of CM cells **A**. Basal protein level of p-ERK, p-AKT and PARP signalling in untreated cell lines. **B**-**D**. Cropped western blots of CRMM1, CRMM2 and CM2005.1 treated with increasing doses of Vemurafenib, Dabrafenib, MEK162 and MK2206 at 24 hrs, showing changes in p-ERK and p-AKT. **E**. To confirm the efficacy of MK2206 at the molecular level, the downstream substrate of AKT, p-PRAS40, was detected after 24 hrs of exposure to MK2206. All experiments were repeated three times, and representative graphs are shown.

Western blot analysis was performed to determine the baseline protein levels of BRAF, p-ERK, ERK, p-AKT and AKT in two *BRAF*-mutant CM cell lines CRMM1 and CM2005.1 and the *NRAS*-mutant cell line CRMM2 (Figure 2A). BRAF is expressed in all three cell lines, with the highest level observed in CRMM1.

**Figure 3:**
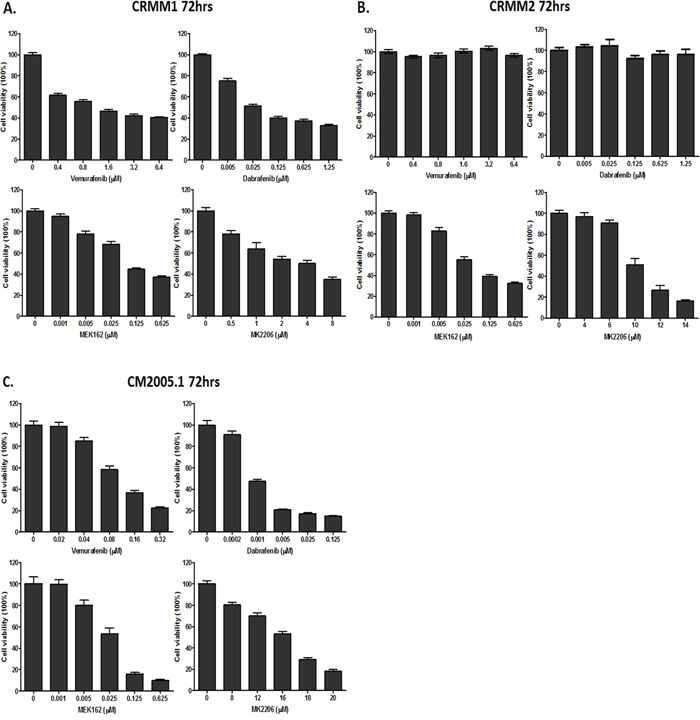
Cell viability assessed by In-cell western assay **A-C**. CRMM1, CRMM2 and CM2005.1 were counted and seeded, and drugs were added one day later at indicated concentrations. Cell viability was measured after 72hrs. All experiments were repeated three times, and the representative data were expressed as mean ± standard error of the mean (SEM).

To determine whether the responses to drugs are pathway specific, we treated different cell lines with BRAFi (Vemurafenib, Dabrafenib), MEKi (MEK162) or AKTi (MK2206) for 24 hours and analyzed the effects on p-ERK and p-AKT levels by western blotting (Table 3). In CRMM1 (Figure 2B), the lowest dose of Vemurafenib (0.4 μM) and Dabrafenib (0.005 μM) already inhibited p-ERK. In contrast, in CM2005.1 (Figure 2D), increasing dosages of Vemurafenib and Dabrafenib decreased p-ERK gradually, while total ERK levels were not suppressed. However, p-ERK expression was increased after BRAFi treatment of the *BRAF* WT CRMM2 cells (Figure 2C). This phenomenon is similar to what has been observed in *BRAF* WT cutaneous melanoma cell lines [30]. The effects on p-ERK levels, combined with cell viability data, indicate that the MAPK pathway plays an important role in growth of CM cell lines. In CRMM1 and CM2005.1, p-AKT was slightly attenuated after BRAFi and MEKi treatment without influencing total AKT, which might be an indirect effect due to the growth inhibition caused by these drugs. In CRMM2, p-AKT was upregulated by BRAFi, but downregulated by MEKi. Low concentrations of MK2206 (CRMM1 0.5 μM, CRMM2 2μM and CM2005.1 4μM) reduced the level of p-AKT, but higher concentrations were needed to suppress cell growth (Figure 3). To investigate whether the AKT pathway was effectively inhibited by MK2206, we determined the p-PRAS40 level, since PRAS40 is a direct downstream target molecule of AKT. As shown in Figure 3E, levels of p-PRAS40 were strongly reduced in all three cell lines upon MK2206 treatment at relative low concentrations, indicating that AKT activity was decreased by MK2206; however, this inhibition was not sufficient to decrease the cell growth of CRMM2 and CM2005.1.

**Table 2:** The sensitivity of conjunctival melanoma cell lines to Vemurafenib, Dabrafenib, MEK162 and MK2206

Cell line	Mutation	Vemurafenib (μM)	Dabrafenib (μM)	MEK162 (μM)	MK2206 (μM)
CRMM1	*BRAF* V600E	0.99±0.07	0.10±0.01	0.09±0.02	4.90±1.25
CRMM2	*NRAS* Q61L	>6.4	>1.25	0.05±0.01	9.67±0.91
CM2005.1	*BRAF* V600E	0.10±0.01	0.003±0.001	0.03±0.01	16.4±1.61

Cells were exposed to the single agent for 72 hrs to assess the cell viability. And the dose-response IC_50_ values were calculated using Compusyn software. Data were presented as the mean ± SD from at least 3 independent experiments.

**Table 3:** Summary of drugs effects on cell proliferation and protein expression *in vitro*

	CRMM1	CRMM2	CM2005.1
Mutations ^a^			
*BRAF* V600E	+	-	+
*NRAS* Q61L	-	+	-
Inhibitiont of cell proliferation ^b^			
BRAFi			
Vemurafenib	+	-	+
Dabrafenib	+	-	+
MEKi			
MEK162	+	+	+
AKTi			
MK2206	+	+	+
Drug effect on phosphorylated protein expression ^c^			
Vemurafenib	p-ERK↓	p-ERK↑	p-ERK↓
Dabrafenib	p-ERK↓	p-ERK↑	p-ERK↓
MEK162	p-ERK↓	p-ERK↓	p-ERK↓
MK2206	p-AKT↓	p-AKT↓	p-AKT↓

^a^ “+” means mutant, and “-” indicates wild type.

^b^ “+” shows cell proliferation was inhibited, while “-” demonstrates cell viability was not influenced.

^c^ “↑” represents phosphorylation of proteins was increased, while “↓” implies phosphorylation of proteins was decreased.

### *In vitro* activity of BRAF, MEK and AKT inhibitors in CM cell lines

Cells were treated with each drug at 5 different concentrations and survival was determined 72 hours later (Figure 3). As shown in Figure 3, Vemurafenib and Dabrafenib inhibited the growth of the *BRAF*-mutant cell lines CRMM1 and CM2005.1, but not of the *NRAS*-mutant cell line CRMM2. MEK162 and MK2206 inhibited growth of all CM cell lines in a dose-dependent manner, although only high concentrations of MK2206 were able to suppress the proliferation of CRMM2 and CM2005.1. Table 2 summarizes the IC_50_ values of each agent for the three CM cell lines. Although CRMM1 and CM2005.1 both harbor the *BRAF* V600E mutation, their sensitivity to BRAFi differed very much. No indications for *PTEN* loss of function were found in the *BRAF* V600E mutated cell lines that could explain this difference. However, we did find a deletion in exon 2 of *PTEN* in the *NRAS* mutant cell line CRMM2.

### G1 arrest and apoptosis after exposure to single drug treatment

**Figure 4:**
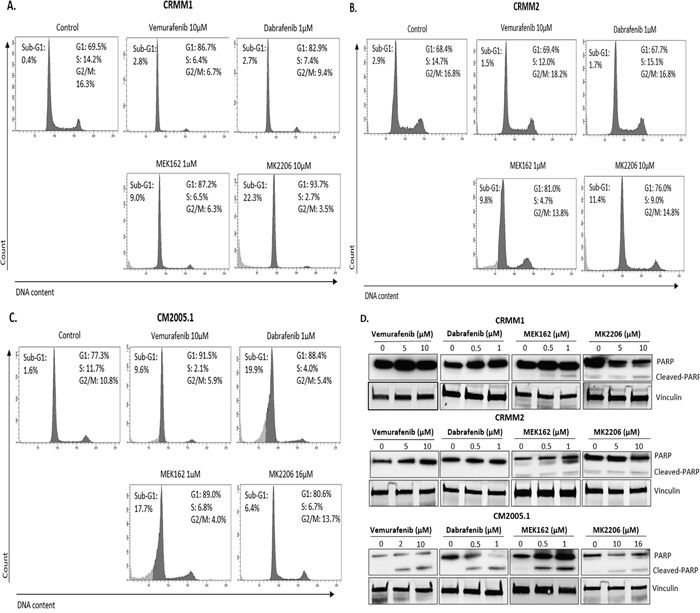
BRAFi, MEKi and AKTi induce cell cycle alteration and apoptosis **A-C**. Representative flow cytometry analysis 72hrs post treatment as indicated. **D**. Cropped western blots of PARP cleavage in cell lines treated with inhibitors at 72hrs.

To obtain more insight into the effects of kinase inhibitors on cell growth and survival, we investigated their impact on cell cycle profiles using flow cytometry (Figure 4A-4C). Treatment with 4 different kinase inhibitors of cell line CRMM1 resulted in strong G1 arrest. MEK162 and MK2206 increased the sub-G1 fraction, indicating that these two drugs were able to induce cell death. As expected, BRAFi did not induce any cell cycle change in CRMM2. Both MEK162 and MK2206 treatment led to G1 arrest, a modest increase in the sub-G1 fraction and a reduced number of S-phase cells (Supplementary Figure S2), although the effect of MK2206 was not very strong. In CM2005.1, G1 arrest and depletion of S-phase cells were found after all drug treatments, although MK2206 treatment did not result in a reduced G2/M phase. Some increase in sub-G1 cells was detected upon all treatments, although it was very modest after MK2206 treatment.

Poly (ADP-ribose) polymerase (PARP) cleavage is often associated with apoptotic cell death and has served as a marker for apoptosis and caspase activity [31]. CRMM1 showed high baseline level of PARP (Figure 2A), while CRMM2 and CM2005.1 showed modest and low PARP expression, respectively. Figure 4D shows that at the indicated concentrations, Vemurafenib and Dabrafenib promoted PARP cleavage in CM2005.1, but not in CRMM1 and CRMM2. MEK162 induced some cleaved PARP in cell line CM2005.1, while PARP cleavage was weak in CRMM1 and CRMM2. MK2206 treatment led to low PARP cleavage in all cell lines.

### Synergistic growth inhibition and cell cycle arrest by combining MEK162 with MK2206

**Figure 5:**
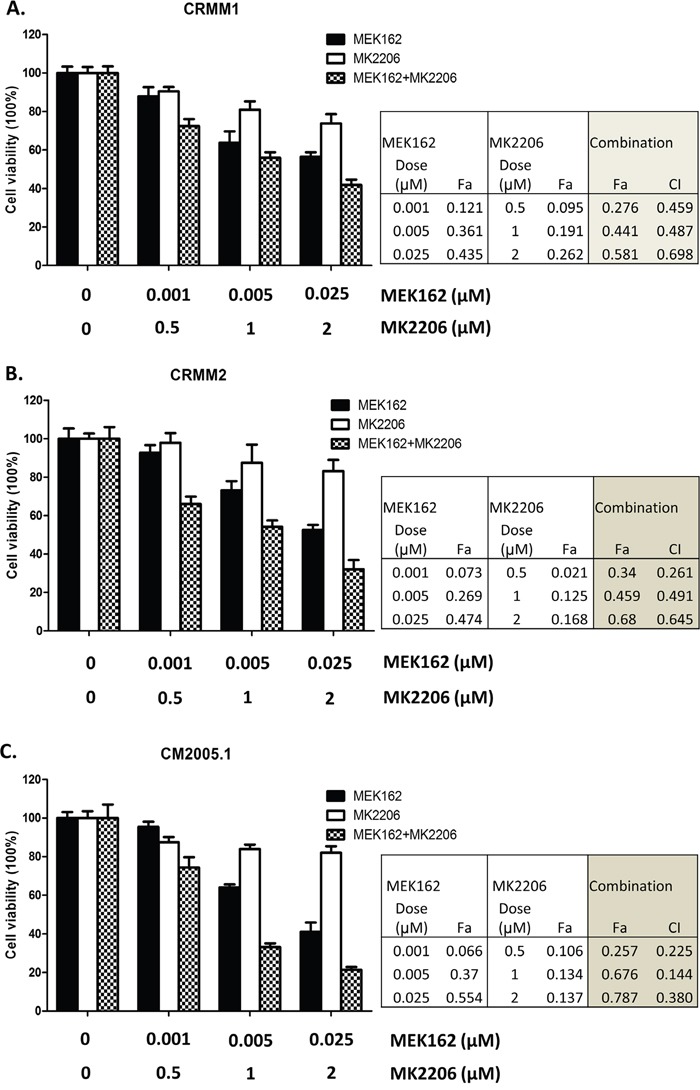
Synergistic growth inhibition using the combination of MEK162 and MK2206 Mono or combination treatments were performed on CRMM1 **A**., CRMM2 **B**. and CM2005.1 **C**. using MEK162 and MK2206 with indicated concentrations. Cell proliferation was measured after 72hrs treatment, and the effects of drug as fraction of control cells were calculated. The combination index (CI), reflecting the extent of synergy or antagonism for two drugs, was obtained for each drug combination. CI<0.9, synergy; 0.9<CI<1.1, additive effect; CI>1.1, antagonism. The experiments were performed three times and representative data were expressed as mean ± SEM.

Treatment of MEKi combined with AKTi has been used to treat *BRAF*-mutant, *BRAF*-WT and *NRAS*-mutant cutaneous melanoma patients [28]. We tested whether combining a MEK and AKT inhibitor would be effective in CM cell lines. We treated CRMM1, CRMM2 and CM2005.1 with varied concentrations of MEK162, MK2206 or the combination of MEK162 and MK2206 for 72 hours, and evaluated cell growth. Synergy studies were performed using the method of Chou [32]. Figure 5 shows that MEK162 and MK2206 were synergistic in all cell lines, with synergy confirmed by CI values. These results suggest that at low concentrations, the two drugs may be used clinically to inhibit cell growth effectively.

**Figure 6:**
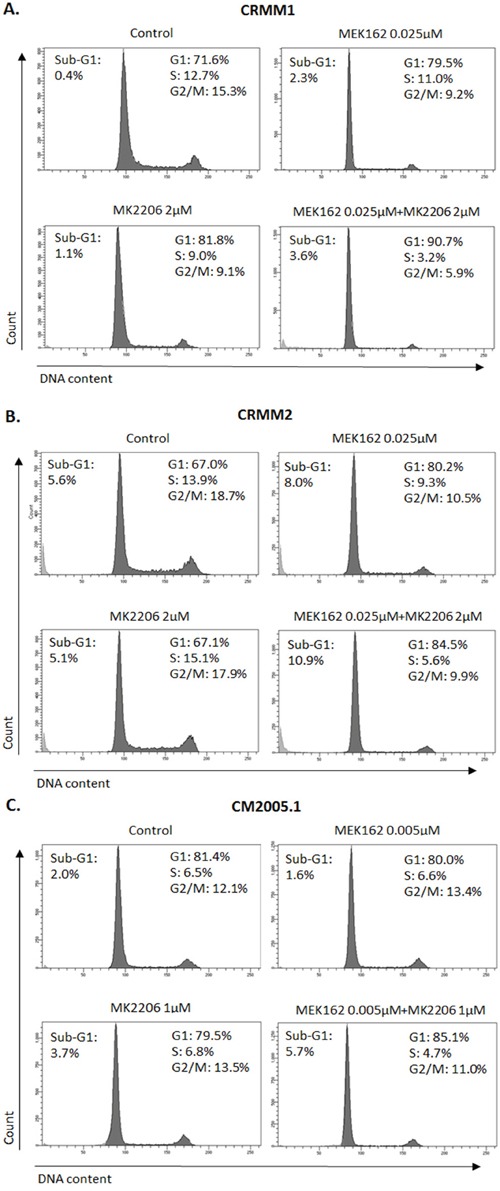
Enhanced G1 arrest induced by MEK162 combined with MK2206 at low concentrations Single or combination treatments were performed in CRMM1 **A**. and CRMM2 **B**. using 0.025μM MEK162 and 2μM MK2206 and in CM2005.1 **C**. using 0.005μM MEK162 and 1μM MK2206. Flow cytometry was analyzed after 72hrs of treatment.

**Figure 7:**
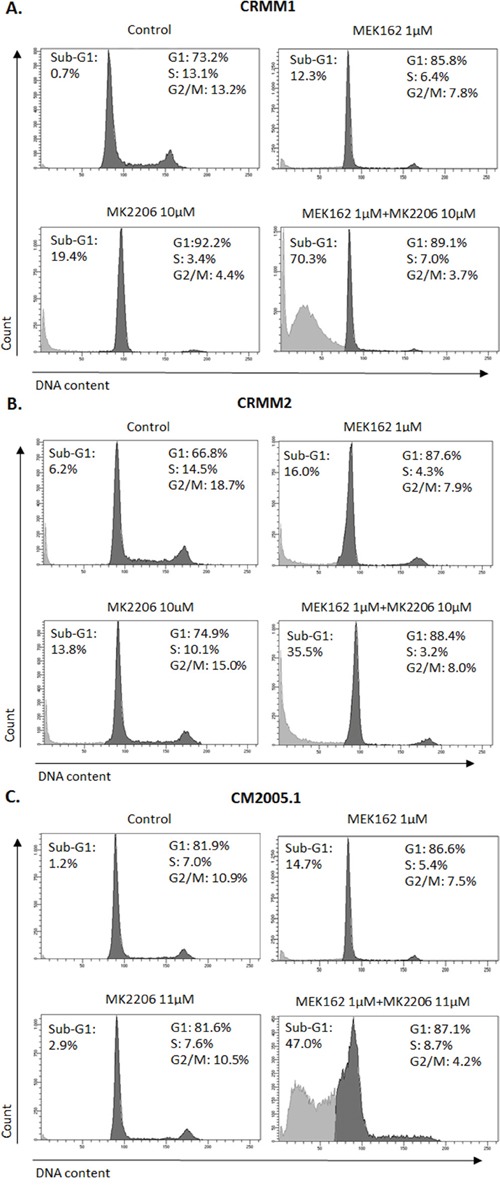
Increased sub-G1 fractions induced by MEK162 combined with MK2206 at high concentrations Mono or combination treatments were performed in CRMM1 **A**. and CRMM2 **B**. using 1μM MEK162 and 10μM MK2206 and in CM2005.1 **C**. using 1μM MEK162 and 11μM MK2206. Flow cytometry was studied after 72hrs of treatment.

To investigate whether combination treatments would inhibit cell cycle progression and induce more cell death than single treatments, we performed flow cytometry. In all cell lines, the combination of MEK162 and MK2206 at low concentrations caused slightly stronger G1 arrest and depletion of S-phase compared to single treatments (Figure 6). The combination of high concentrations induced stronger sub-G1 fractions than single agents (Figure 7).

## DISCUSSION

Uveal melanomas, in contrast to conjunctival melanomas, lack *BRAF* or *NRAS* mutations but frequently have mutations in *GNAQ*, *GNA11*, *BAP1*, *SF3B1* or *EIF1AX* [33]. To date, the reports describing the impact of kinase pathways in conjunctival melanoma are limited. Our results show that MAPK and AKT-signaling pathways are activated in both benign, premalignant and malignant conjunctival melanocytic lesions. In our series, *BRAF* V600E mutations occur in 19% of nevi and 26% of CM, but not in PAM, which confirms previous results [16]. Likewise, *BRAF* V600E mutations have been described to be less frequent in lentigo maligna lesions of the skin which show a comparable lentiginous histopathology as PAM [34]. Furthermore, similar to the findings in melanocytic nevi and cutaneous melanoma [35, 36], we report that *BRAF* mutations do not determine the tumor's ERK phosphorylation status. It is unlikely that technical problems such as tissue fixation and the high temperature used for antigen retrieval impaired the staining of p-ERK, because positive p-ERK cells were abundant in positive control tissues (data not shown). Recently, loss of function mutations in NF1 have been described in a high percentage of cutaneous melanoma. NF1 suppression can lead to increased RAS activation in melanoma, which may explain the lack of correlation between ERK phosphorylation and BRAF mutational status [37, 38].

Although p-ERK and p-AKT are regarded as being predominantly situated in the cytoplasm, the phosphorylation of ERK and AKT in the nucleus is essential for nuclear translocation and activation of transcription factors [39, 40]; we therefore scored the staining in nuclei as well as in the cytoplasm. Our data show that, in general, p-ERK and p-AKT are indeed expressed in both nuclei and cytoplasm among all diagnostic groups of conjunctival melanocytic lesions, although with variable percentages. These results indicate that MAPK and PI3K/AKT pathways are both potential targets for pharmacologic therapies in CM.

To investigate the effect of different drugs on cell proliferation and cell death, we tested BRAF, MEK and AKT inhibitors on three CM cell lines. Vemurafenib and Dabrafenib are selective BRAF inhibitors, and have been approved by the FDA as single agents for treatment of metastatic cutaneous melanoma. MEK162 and MK2206 target MEK and AKT respectively. Although CRMM1 and CM2005.1 both harbor a *BRAF* V600E mutation, their sensitivity to BRAFi differed greatly: CRMM1 required a much higher dose of Dabrafenib to inhibit cell growth compared to CM2005.1. This could not be explained by loss of function in *PTEN*. Additionally, we demonstrate that BRAFi induced apoptosis of CM2005.1 cells. The cell cycle analysis indicates that BRAFi inhibited cell growth of CRMM1 by inducing a G1 arrest. In contrast, Vemurafenib and Dabrafenib did not inhibit cell proliferation of CRMM2, and they led to an increased p-ERK expression. This finding is in agreement with other preclinical studies in other malignancies, in which selective BRAFi stimulated cell growth and ERK phosphorylation in *BRAF* WT and *NRAS* mutant cutaneous melanoma cell lines [41–43]. The mechanism of the detrimental effects is through paradoxical-activation of RAS and receptor tyrosine kinases, which subsequently cause MAPK pathway hyperactivation through CRAF [44].

MEK162 inhibits the downstream RAS/RAF pathway target MEK, thereby decreasing p-ERK levels and suppressing cell growth. We did not find other studies that have treated CM cells with this novel drug. Proliferation of all three cell lines was inhibited through downregulation of p-ERK upon MEK162 treatment. Furthermore, the data of cell cycle profiles and PARP cleavage show that in addition to cytostatic effects, MEK162 alone can prompt apoptosis, regardless of *BRAF* or *NRAS* mutations. Importantly, IC_50_ values for cell culture are much lower than known plasma levels of MEK162 in patients (0.6-1 μM). However, similar to BRAFi, the disadvantage is that constitutive activation of the MAPK pathway can frequently lead to cross-talk with other signal transduction pathways, enabling melanoma cells to escape from MEK inhibition [45]. Current MEKi have more clinical side effects than Vemurafenib and Dabrafenib.

The PI3K/AKT signal transduction pathway is considered a potential co-target for *BRAF* and *NRAS* mutant cutaneous melanoma [22]. To the best of our knowledge, we are the first to attempt AKT inhibition in CM *in vitro*. Our results show that MK2206 inhibited proliferation in all CM cell lines, but at variable doses, and independent of their effect on p-AKT and p-PRAS40, indicating that the growth inhibitory effect induced by MK2206 is not specific or at least partly caused by off-target effects.

With the clinical availability of PI3K, AKT and mTOR inhibitors, a number of trials are now ongoing in cutaneous melanoma. Our studies show that combining MEK162 with MK2206 has a synergistic inhibitory effect on proliferation of three CM cell lines, regardless of their sensitivity to the individual agents. Mutations in the G-α-proteins *GNAQ* and *GNA11* that occur in 91% of uveal melanoma also activate MAPK and PI3K/AKT pathways, which implies that the combination of MEK and AKT inhibitors may also be profitable for this patient group. Future studies on cutaneous and uveal melanoma cell lines are therefore warranted to evaluate the efficacy of combination treatment of MEK and AKT inhibitors. In clinical treatment, one may argue that multi-signal pathway blockades generate more side effects than individual kinase inhibitor. On the other hand, we have shown that combined treatment could be effective at considerably lower drug dosages and potentially ameliorate detrimental side effects.

In summary, our data suggest that the ERK and AKT are constitutively activated in all conjunctival melanocytic lesions. Our *in vitro* study shows some growth inhibition and cytotoxic effect of kinase inhibitors and provides a rationale for the clinical application of these compounds in CM. Our results indicate that co-inhibition of the MAPK and AKT pathways may improve their efficacy. Clinical studies are now indicated to test the efficacy of MAPK and AKT inhibitors in metastatic CM.

## MATERIALS AND METHODS

### Patients and samples

Paraffin-embedded tissue specimens were retrieved from the archive of the Department of Pathology of the Erasmus University Medical Center from patients with a conjunctival melanocytic lesion diagnosed between 1987 and 2013. The Institutional Review Board of the Erasmus University Medical Center waived the need for approval because of the retrospective and non-interventional character of the study. The study adhered to the tenets of the Declaration of Helsinki. Two hundred and seventy-eight samples obtained from 131 conjunctival lesions of 129 patients contained enough material for the study. In our patient cohort, CM were derived from PAM (59.5%, *n*=25), de novo (28.6%, *n*=12) and nevi (11.9%, *n*=5). All melanomas described were invasive. The metastatic rate was 21.4% (*n*=9). Out of 18 PAM with atypia, 8 had severe atypia and 10 moderate atypia. No cases with minor atypia were included. The diagnosis was reviewed and the original slides were assessed according to the AJCC criteria. Because of the high recurrence rate, some patients underwent multiple surgeries.

### Construction of tissue microarray (TMA) samples

Carefully selected cores of 1 mm in diameter were taken from paraffin-embedded conjunctival tumor samples and assembled in a grid pattern into seven tissue microarrays. Positive control samples consisted of brain, liver, tonsil, heart, lung, gut and skin. The 4-μm sections were cut from TMA and were stained with hematoxylin and eosin (HE), and anti-Melan A to confirm the presence of the expected tissue histology within each tissue core. Additional sections were cut for further immunohistochemical analysis.

### Immunohistochemistry (IHC)

We assessed the expression of p-ERK, p-AKT Ser473, p-AKT Thr 308 and BRAF V600E. Immunohistochemistry was performed with an automated staining system (Ventana BenchMark ULTRA, Ventana Medical Systems Inc., Tucson, AZ, USA) using the alkaline-phosphatase method for all antibodies and a red chromogen. Briefly, following deparaffinization and heat-induced antigen retrieval for 60 minutes, the tissue sections were incubated with primary antibody anti-MAP kinase diphosphorylated ERK 1/2 (1:1000; M8159, Sigma-Aldrich, St. Louis, MO, USA), p-AKT Ser473 (sc-135651; 1:25, Santa Cruz Biotechnology; Dallas, TX, USA), p-AKT Thr 308 (sc-135650, 1:50, Santa Cruz Biotechnology), BRAF-V600E (26039, 1:50, NewEast Biosciences, Malvern, PA, USA) or Melan A (clone A103, Ventana) for 1 hour at 36°C. A subsequent amplification step was followed by incubation with hematoxylin II counter stain for 8 minutes and then bluing reagent for 8 minutes according to the manufacturer's instructions (Ventana). The immunostained TMAs were analyzed with light microscopy (Leica DM 300, Leica, Eindhoven, the Netherlands) by two different observers at different time points. The samples were scored using the immunoreactive scoring (IRS) method, described by Remmele and Stegner [46]. The IRS evaluates the staining intensity and the proportion of positive cells, which results in two scores: an intensity score (IS) and a proportion score (PS). The value of the IS varies from 0 to 3 points and the value of the PS varies from 0 to 4 points. Multiplying these scores yields the IRS (0 - 12 points). The representative pictures of staining were taken with an Olympus DP25 camera, and acquired by Olympus CellSens Entry 1.9 software. The immunohistochemical markers stained the cytoplasm of the melanocytes. Some markers also stained the nucleus. For these markers, the staining of the cytoplasm and nucleus were analyzed and scored separately.

### Reagents

Vemurafenib (PLX4032, S1267), Dabrafenib (GSK2118436, S2807), MEK162 (ARRY-162, S7007) and MK2206 (S1078) were purchased from Selleck Chemicals (Huissen, The Netherlands). All drugs were dissolved in dimethyl sulfoxide (DMSO) to reach a stock concentration of 10 mM. For *in vitro* tests, the stock solution was diluted in the indicated fresh medium.

### Cell lines and cell culture

CM cell lines CRMM1 and CRMM2 were established by G. Nareyeck, Essen, Germany [47], and kindly provided by M. Madigan, Sydney, Australia. The cells were cultured in F-12K nutrient mixture, Kaighn's modification (Gibco, Life Technologies, The Netherlands), with added heat-inactivated 10% fetal bovine serum (FBS, Greiner Bio-one, The Netherlands) and 1% Penicillin/Streptomycin (Gibco). CM2005.1 was created by S. Keijser, LUMC, Leiden, The Netherlands [48]. These cells were grown in RPMI 1640 Dutch modified media (Gibco) supplemented with 10% FBS (Greiner Bio-one), 1% GlutaMAX and 1% Penicillin/Streptomycin (Gibco). Our previous studies [49, 50] have shown that CRMM1 and CM2005.1 harbor a *BRAF* V600E mutation, and CRMM2 contains an *NRAS* Q61L mutation. These mutations were confirmed at Erasmus University Medical Center, Rotterdam in May 2016 by next generation sequencing (NGS). Inactivating molecular changes in *PTEN* were tested by NGS as decribed before [51].

### Cell line authentication

Short tandem repeat analysis was performed using the AmpFLSTR® Identifiler™ PCR Amplification Kit (Life Technologies, UK) based on the procedure recommended by *The International Cell Line Authentication Committee* (ICLAC) in Baseclear (Baseclear, Leiden, The Netherlands) in April, 2014, setting the standard for these three cell lines [50]. They were confirmed at Erasmus University Medical Center, Rotterdam in October 2014.

### Immunoblotting

Cells were lysed in M-PER Mammalian Protein Extraction Reagent (78501, Thermo Scientific, OH, USA), supplemented with protease and phosphatase inhibitors (78415 and 78420, Thermo Scientific). Proteins (15-25 μg total protein lysates) were separated on Mini-PROTEAN TGX Precast Gels (4-15%, Bio-Rad) using the Mini-PROTEAN Tetra system (Bio-Rad, Hercules, CA). Proteins were blotted onto polyvinylidene difluoride (PVDF) membranes (Immobilon-P, Millipore, Billerica, MA, USA) using the Trans-Blot Turbo System (Bio-Rad). Subsequently, the membranes were incubated with the appropriate primary antibodies and IRdye-680 or IRdye-800 dyes (LI-COR), and analyzed with the Odyssey Infrared Imaging System (LI-COR). Signal intensity was analyzed with Odyssey 3.0 software. Alternatively, membranes were incubated with HRP-conjugated secondary antibodies, and the bands were visualized by chemoluminescence (West Dura, Pierce Biotechnology, Rockford, IL, USA) and exposed to X-ray films (Fuji Super RX, Japan). Antibodies are listed below: BRAF (1:1000; ab33899, Abcam, Cambridge, UK); anti-MAP kinase diphosphorylated ERK 1/2 (1:1000; M8159, Sigma-Aldrich); p44/42 MAPK (ERK1/2) (1:1000; 4695, Cell Signaling Technology (CST), Leiden, The Netherlands); p-AKT (Ser473) (1:1000; 4060, CST); AKT (pan) (1:2000; 2920, CST); PARP (1:1000; 9542, CST); p-PRAS40 (Thr246) (1:1000; 2640, CST), PRAS40 (1:1000; 2610, CST) and Vinculin (1:1000; V9131, Sigma-Aldrich).

### Cell proliferation assay

Cells were seeded in triplicate in 96-well plates at a density of 2500 (CRMM1 and CRMM2) or 5000 (CM2005.1) cells per well, in a total volume of 100 μl medium. The next day, the media were refreshed and cells were treated with drugs at various concentrations. Cell survival was determined 72 hours later by an In-cell western assay (Supplementary Figure S1): after removing the medium, the cells were fixed in 4% formaldehyde and incubated with DRAQ5, a far-red fluorescent DNA dye (1:8000, DR50050, Biostatus Ltd., UK) for 1 hour. After washing with 0.1% Tween-PBS (phosphate-biffered saline) buffer, the plates were scanned with an Odyssey Infrared Imaging System (LI-COR, Leusden, The Netherlands). Odyssey 3.0 software was used to quantify the signal intensity. IC_50_ values were calculated on the basis of the growth inhibition curves and determined with CompuSyn software [32]. For synergy studies, drug effects were evaluated as “affected fraction” of treated versus untreated cells [52]. Dose-effect analyzes and Combination Index (CI) were calculated using CompuSyn. CI reflects the extent of synergy or antagonism for two drugs: CI<0.9, synergy; 0.9<CI<1.1, additive effect; CI>1.1, antagonism.

### Flow cytometry

Cells were harvested, washed in PBS and fixed in ice-cold 70% ethanol and stored at -20°C. Subsequently, cells were washed with 2%FBS/PBS and suspended in 2%FBS/PBS containing 50 μg/ml propidium iodide (PI) and 50 μg/ml RNase. Flow cytometry was performed in the BD LSR II system (BD Biosciences, Sparks, MD, USA) and the data were analyzed with FACSDiva software (BD).

### Statistical analysis

Statistical analyses were performed with SPSS version 22.0 software (SPSS, Inc., Chicago, IL, USA). The Pearson chi-square test was used to compare the protein expression *in vivo*. Two-tailed *P* values equal to or below 0.05 were considered to be statistically significant. The plots of cell proliferation and cell cycle profiles were drawn with GraphPad Prism 5 software (La Jolla, CA, USA).

## SUPPLEMENTARY MATERIALS FIGURES


