# Supplementary material for: Targeting of the MAPK and AKT pathways in conjunctival melanoma shows potential synergy
Source: Oncotarget. 2016 Jul 22;8(35):58021–36. doi: 10.18632/oncotarget.10770 (PMC5601630; doi:10.18632/oncotarget.10770)
Supplement: Supplementary file 1 [file oncotarget-08-58021-s001.pdf]

Targeting of the MAPK and AKT pathways in conjunctival melanoma shows potential synergy

SUPPLEMENTARY FIGURES

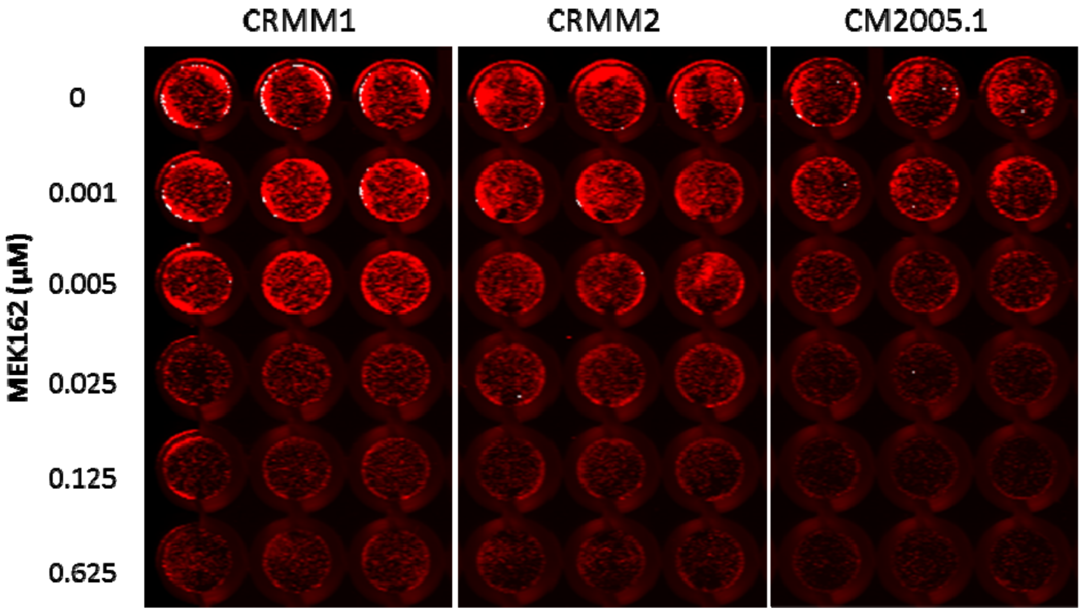

Supplementary Figure S1: In-cell western assay for CM cell lines treated with MEK162 for 72 hrs. MEK162 inhibited cell growth of CRMM1, CRMM2 and CM2005.1. Cells were seeded in triplicate in a 96-well plate for 24 h, and then MEK162 (0.001-0.625µM) was added from top line to bottom. In-cell western assay with DRAQ5 stained the DNA content (red) in each well, and the intensity was measured to interpret the cell growth.

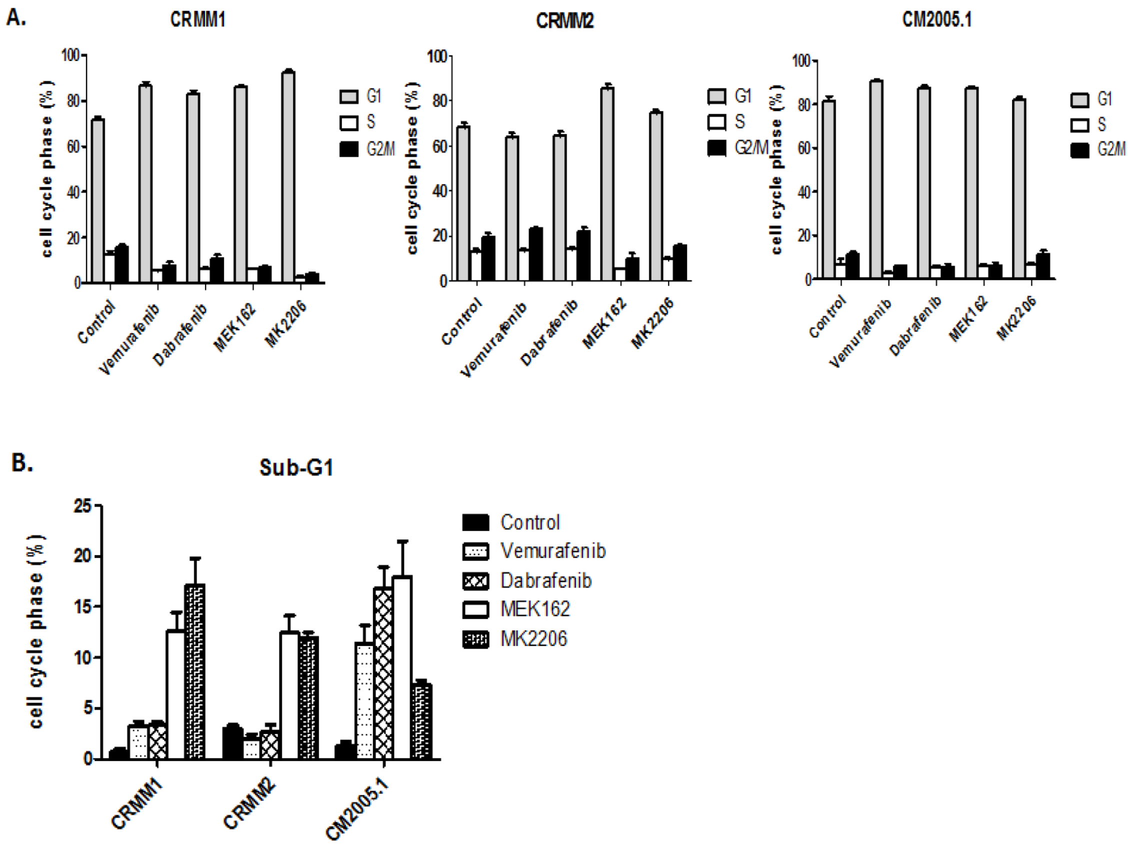

Supplementary Figure S2: BRAFi, MEKi and AKTi induce cell cycle alteration. A. The statistical analysis of cell cycle profiles from three independent experiments. Bars indicate the mean and SEM. **B.** Evaluation of Sub-G1 fractions. Bars represent the mean and SEM of three independent experiments.
